# CMR myocardial texture analysis tracks different etiologies of left ventricular hypertrophy

**DOI:** 10.1186/1532-429X-18-S1-O82

**Published:** 2016-01-27

**Authors:** Rebecca Schofield, Balaji Ganeshan, Rebecca Kozor, Arthur Nasis, Raymond Endozo, Ashley Groves, Charlotte Manisty, James C Moon

**Affiliations:** 1 Cardiology, Barts Heart Centre, London, UK; 2 grid.83440.3b0000000121901201Institute of Nuclear Medicine, University College London, London, UK; 3 MonashHeart, Melbourne, NSW Australia

## Background

Defining the underlying etiology of heart muscle disease is important. Tissue characterisation with late gadolinium enhancement (LGE) and the newer parametric mapping technologies aim to do this. Texture analysis (TA) is another tool that may help. Macroscopic heterogeneity in texture on diagnostic imaging, both at and beyond that appreciated by the human eye, are known to track microscopic histological characteristics in other branches of medicine (computed tomography, oncology). We hypothesised that TA could be 1. translated to CMR cine images and quantify and detect image signal heterogeneity, and 2. the measured signal would track underlying disease etiology.

## Methods

185 cases were studied: (50 HCM, 52 amyloid, 68 aortic stenosis (AS) and 15 healthy controls). All images were pre-contrast and considered free of susceptibility artefact or any gating abnormalities. For each only the end-diastolic frame of a mid short axis cine was studied. Analysis was using TexRAD commercial research software (TexRAD Ltd, http://www.texrad.com, part of Feedback Plc, Cambridge, UK). The analysis comprised firstly selective scale image filtration to extract features corresponding to fine, medium and coarse texture scales, and secondly texture quantification by histogram analysis quantified using 6 parameters: mean intensity, standard-deviation (SD), entropy, mean of positive pixels (MPP), skewness, and kurtosis.

## Results

**Figure 1:**
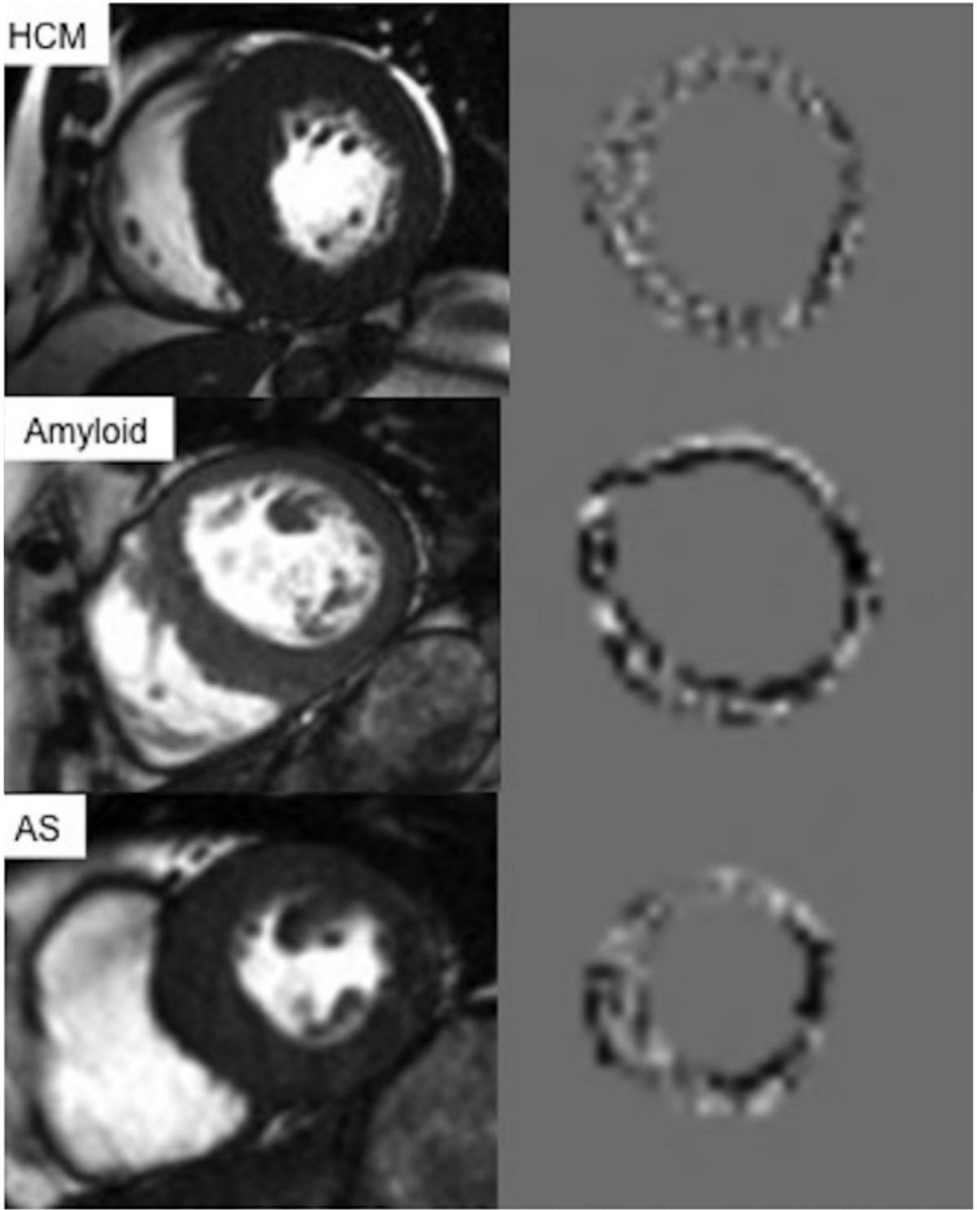
**A CMR single mid-ventricual short-axis diastolic frame and the corresponding CMR-TA comprising fine filtered whole ventricular wall texture map for 3 diseased cases: hypertropic cardiomyopathy (HCM), amyloid and aortic stenosis (AS)**.

Figure 1 illustrates the CMR-TA process within 3 diseased cases. There were significant differences between HCM, amyloid, AS and healthy controls. These were predominantly at the fine and medium texture scales for most quantifiers (mean intensity, SD, entropy and MPP) across the whole, septum, anterior, lateral and inferior segments of the ventricular wall (p < 0.001). Specifically, these texture-quantifiers followed a significant trend were SD, entropy and MPP were lowest, whilst mean intensity was highest for HCM followed by amyloid, AS and finally healthy controls. Amongst the HCM patients the above texture parameters significantly differentiated those with and without LGE (p = 0.015).

## Conclusions

Texture analysis of conventional CMR cine images may complement conventional myocardial tissue characterisation in LVH - here detecting changes that appear to track etiology and LGE scar.

